# Exceptional response to brigatinib following alectinib failure in a patient with *ALK* fusion-positive duodenal carcinoma

**DOI:** 10.1007/s13691-025-00745-2

**Published:** 2025-02-10

**Authors:** Akinori Sasaki, Sayaka Chihara, Risa Okamoto, Takayuki Yoshino, Yoshiaki Nakamura

**Affiliations:** 1 Department of Gastroenterology, Tokyo Bay Urayasu Ichikawa Medical Center, Urayasu, Chiba Japan; 2 Department of Oncology, Tokyo Bay Urayasu Ichikawa Medical Center, Urayasu, Chiba Japan; 3 Department of Gastrointestinal Oncology, National Cancer Center Hospital East, Kashiwa, Japan

**Keywords:** Duodenal cancer, *EML4*-*ALK* rearrangement, Secondary ALK mutation, Brigatinib, ctDNA sequencing

## Abstract

Patients with advanced duodenal carcinoma typically have a poor prognosis due to limited practical chemotherapy options. While studies on genotype-directed therapy in patients with duodenal carcinoma is progressing, clinical data assessing the efficacy of molecularly targeted therapy remains scarce. We report the case of a 65-year-old woman diagnosed with anaplastic lymphocyte kinase (*ALK*) fusion-positive advanced duodenal carcinoma. The patient had been treated with alectinib for approximately 2 years for *ALK*-positive duodenal carcinoma but developed progressive liver metastases, indicating alectinib failure. During the disease progression, circulating tumor DNA (ctDNA) sequencing revealed the emergence of *ALK* L1196M mutation, which demonstrated sensitivity to brigatinib. After switching to brigatinib, marked shrinkage of liver metastases was observed. The patient maintained brigatinib treatment for 7 months until tumor progression. This is the first report demonstrating the efficacy of brigatinib after alectinib failure in a patient with duodenal carcinoma harboring *ALK* fusion. Furthermore, this case suggests that ctDNA sequencing can detect specific acquired mutations and help expand optimal treatment options for patients.

## Introduction

Small intestine cancer, including duodenal carcinoma, is a rare disease with a global incidence of less than 1 per 100,000 people [1]. Nearly half of patients with duodenal carcinoma were diagnosed at stage IV [2]. Although oxaliplatin-based systemic therapy is indicated for the patients with advanced duodenal carcinoma, the available evidence is insufficient to support the use of systemic chemotherapy [3].

Multigene panel-based comprehensive genomic profiling (CGP) has been approved and is widely utilized to select treatment for patients with malignant solid tumors. CGP facilitates the identification of patients with targetable alterations who may be eligible for targeted therapies. Targetable gene alterations, including *BRAF*, *ERBB2*, and *PIK3CA*, can also be detected in small intestinal cancers, such as duodenal carcinoma [4]. Although rare, anaplastic lymphoma kinase (ALK) alterations, which are usually detected in non-small cell lung cancer (NSCLC), have also been identified in small intestinal cancer [4]. In addition circulating tumor DNA (ctDNA) analysis is a useful method to detect genomic alterations in tumor cells throughout the body and to identify concurrent resistance mechanisms that may be missed by single-lesion tumor biopsies [5–7].

We previously reported the first case of a patient with *ALK* fusion-positive duodenal carcinoma that responded to alectinib [8]. And this time, we experienced a case of *ALK* fusion duodenal carcinoma that developed resistant after initially responding to alectinib. Following disease progression on alectinib, ctDNA testing revealed the emergence of a secondary *ALK* L1196M mutation. The patient subsequently demonstrated a response to brigatinib. The patient provided informed consent for the presentation of the clinical information in an anonymized form.

## Case

**Fig. 1:**
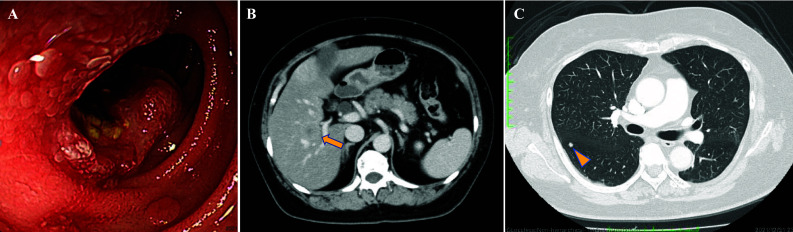
Endoscopic findings and computed tomography images at the time of diagnosis. **A** Endoscopic findings of the duodenum reveal a type 2 tumor on the descending part. **B** Abdominal computed tomography shows liver metastasis (long arrow). **C** Chest computed tomography reveals lung metastasis (arrow head)

**Fig. 2:**
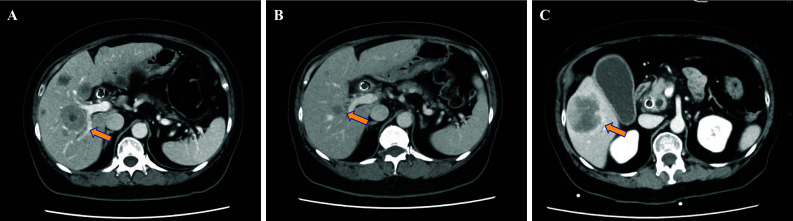
Abdominal computed tomography images during treatment with alectinib. **A**, **B** Abdominal computed tomography showed increased tumor volume of liver metastases 4 weeks after modified FOLFOX6 therapy (A, long arrow) and significant shrinkage of liver metastases 6 weeks after the initiation of alectinib (B, long arrow). **C** Abdominal computed tomography showed the progression of liver metastases 2 years after the alectinib (long arrow)

A 65-year-old female patient was referred to Tokyo Bay Urayasu Ichikawa Medical Center with epigastric pain and weight loss for several weeks. She was diagnosed with stage IV duodenal carcinoma having multiple lymph nodes, liver metastases and lung metastases (Fig. 1). As previously reported [8], tissue-based CGP test of the primary tumor detected echinoderm microtubule-associated protein-like 4 (*EML4*)-*ALK* fusion, as well as alterations in *CDKN2A*/*B* and *TP53* P151R. Treatment with alectinib was initiated following the first-line treatment with modified FOLFOX6. The initial CT scan after administration of alectinib showed a significant tumor reduction in lymph nodes and liver metastases (Fig. 2A and 2B). Treatment with alectinib continued for approximately 2 years without evidence of tumor progression. However, the patient developed resistance to alectinib treatment, as evidenced by progression of liver metastases (Fig. 2C).

**Fig. 3:**
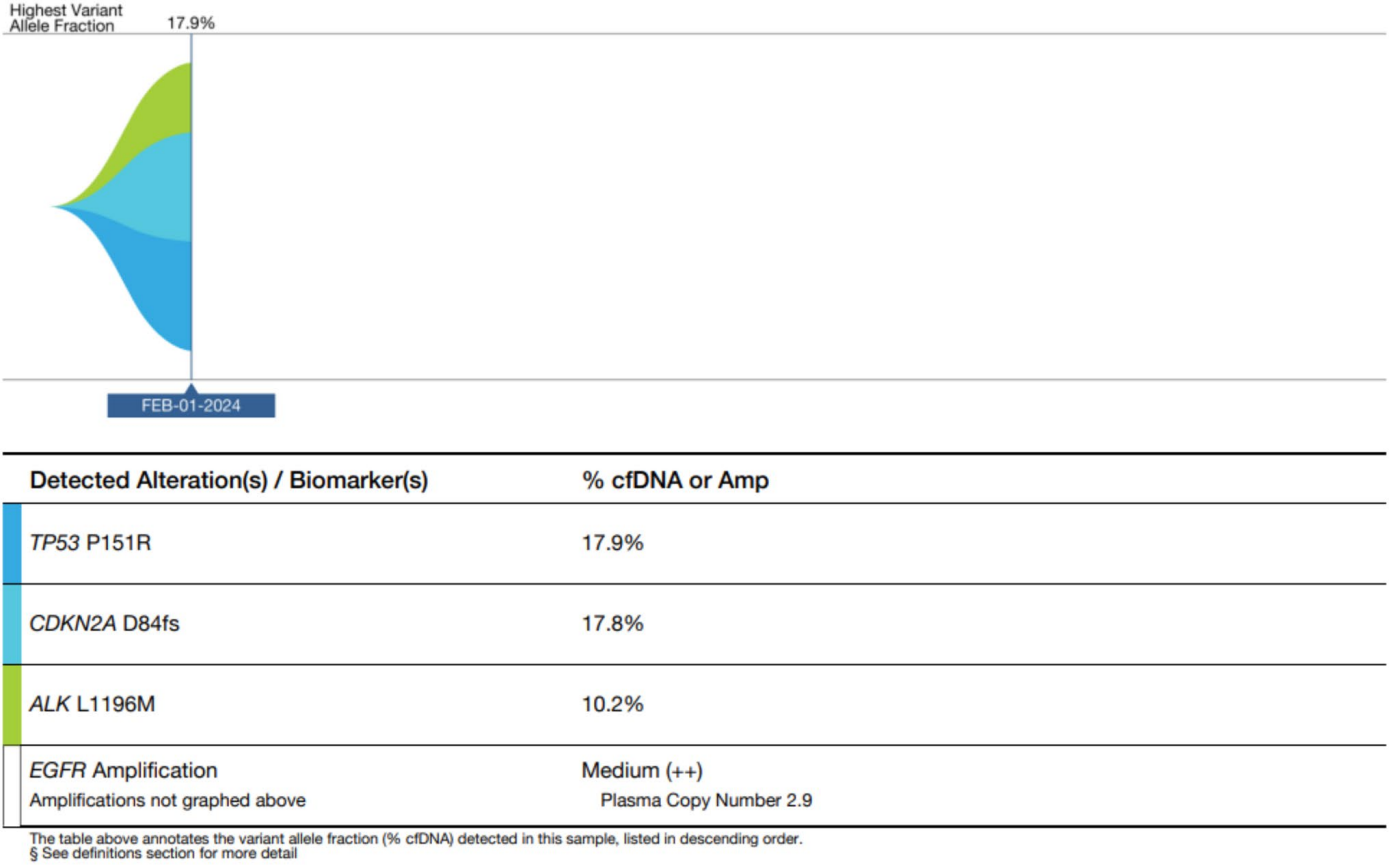
Guardant360 Tumor Response Map. ctDNA sequencing following alectinib failure identified an *ALK* L1196M mutation secondary ALK mutation

**Fig. 4:**
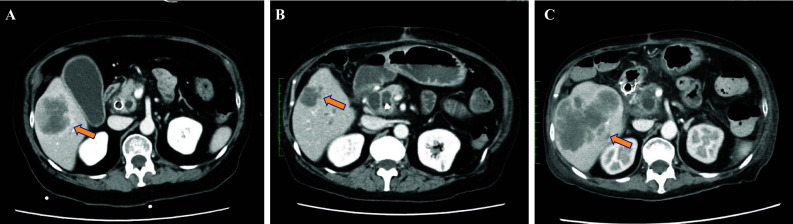
Abdominal computed tomography images during treatment with brigatinib. **A**, **B** Abdominal computed tomography showed the progression of liver metastases 2 years after the alectinib (**A**) and significant shrinkage of liver metastases 6 weeks after the brigatinb (**B**). **C** Abdominal computed tomography showed the progression of liver metastases 7 months after the brigatinb

The patient participated in a ctDNA-based profiling study (GOZILA Study, UMIN-ID: 000029315) and underwent ctDNA sequencing using the Guardant360 assay (Guardant Health, Redwood City, CA) to identify drug-resistant alterations and determine the subsequent treatment regimen. ctDNA sequencing following alectinib failure identified an *ALK* L1196M mutation that was not detected in the initial tissue-based CGP test (Fig. 3). A previous study investigated sensitivity of different ALK inhibitors in Ba/F3 cells with various *ALK* mutations. It showed that the cell harboring the ALK L1196M mutation were resistant to some ALK inhibitors, including alectinib, crizotinib, lorlatinib, and entrectinib, while brigatinib, ceritinib, and gilteritinib demonstrated effective IC50 values against this variant [9]. Based on these ctDNA sequencing results, brigatinib (180 mg daily), a second-generation ALK inhibitor, was initiated. The patient tolerated the treatment without any adverse events. Initial evaluation of the therapeutic effects of brigatinib by CT scan 2 months after treatment initiation revealed significant shrinkage of liver metastases, qualifying as a partial response according to the Response Evaluation Criteria in Solid Tumors version 1.1 (Fig. 4A,B). The patient maintained brigatinib treatment for 7 months without evidence of tumor progression. However, CT scan at 7 months showed progression of liver metastases (Fig. 4C), leading to discontinuation of brigatinib and transition to palliative care.

## Discussion

We present a patient with *ALK* fusion-positive duodenal carcinoma treated with brigatinib following alectinib failure. The patient achieved a partial response to brigatinib after developing an acquired resistance mutation to alectinib. While the efficacy of drugs such as lorlatinib has been documented in ALK-positive NSCLC following progression on second-generation ALK inhibitors [10–12], this represents the first such case in duodenal carcinoma. In our case, ctDNA analysis using Guardant360 during disease progression revealed the emergence of new *ALK* L1196M mutation, leading to the decision to administer brigatinib.

*ALK* rearrangement has been observed in 3–5% of patients with NSCLC [13]. It has also been identified in other cancer types, including non-Hodgkin lymphoma, breast cancer, and colorectal cancer [14, 15]. In addition, there have been reports of *EML4*-*ALK* fusion in small intestine cancer, albeit in exceedingly rare instances, accounting for a mere 0.3% [4]. The sole case previously reported demonstrated the efficacy of an ALK inhibitor in a patient with duodenal carcinoma harboring *ALK* fusion [8].

ALK inhibitors, including alectinib, have demonstrated a pronounced effects on NSCLC exhibiting *ALK* fusions. In patients with treatment-naïve ALK-positive NSCLC, the median progression-free survival with alectinib was 34.8 months, and the overall survival had not yet been reached [16]. Despite the considerable therapeutic efficacy of ALK inhibitors, resistance mechanisms can emerge, leading to treatment failure. These resistance mechanisms are classified as ALK-dependent and non-ALK-dependent [17]. Non-ALK-dependent mechanisms include bypass activation, such as emergence of *MET* and *KRAS* alterations, and histologic transformation. On the other hand, ALK-dependent mechanisms primarily involve *ALK* kinase domain resistance mutations and increased copy numbers of *ALK* rearrangements. Secondary mutations in the *ALK* kinase domain, including G1202R, L1196M, and G1269A, have been documented [18]. Notably, the *ALK* G1202R mutation is frequently observed following treatment with second-generation ALK inhibitors [19].

Lorlatinib, a third-generation ALK inhibitor, is recommended for ALK-positive NSCLC that has become resistant to alectinib [10]. While lorlatinib can overcome most acquired resistance mutations, including *ALK* G1202R within the *ALK* kinase domain [19], not all patients undergo mutation testing when they develop resistance to alectinib in clinical practice. This is significant because some secondary mutations, such as *ALK* L1196M, which may emerge following alectinib resistance, are known to be resistant to lorlatinib treatment.

ctDNA genomic profiling enables the identification of druggable genomic alterations and expands treatment options without tumor biopsies [5]. Furthermore, ctDNA analysis can identify resistance mechanisms following treatment failure. In our case, ctDNA analysis using Gurdant360 after alectinib resistance identified an acquired *ALK* L1196M mutation. Consequently, brigatinib was selected over lorlatinib, achieving a treatment response for 7 months. Dynamic monitoring of resistance mechanisms during ALK inhibitor treatment facilitates optimal survival benefits to patients and represents a crucial step toward precision medicine. At this time, it is not possible to perform multiple CGP tests in actual clinical practice, it is hoped that sequential CGP tests will be approved in future.

In conclusion, to our knowledge, this is the first reported case of a response to brigatinib treatment following alectinib failure in a patient with *ALK* fusion-positive duodenal carcinoma. Despite eventual disease progression, the patient maintained stable disease for 7 months on brigatinib. ctDNA sequencing proved instrumental in identifying a secondary *ALK* mutation, enabling an effective treatment strategy. As this is a case report, further research is required to develop more effective treatment strategies.

## Data Availability

Not applicable.
